# Intracellular Shuttle: The Lactate Aerobic Metabolism

**DOI:** 10.1100/2012/420984

**Published:** 2012-04-19

**Authors:** Rogério Santos de Oliveira Cruz, Rafael Alves de Aguiar, Tiago Turnes, Rafael Penteado Dos Santos, Mariana Fernandes Mendes de Oliveira, Fabrizio Caputo

**Affiliations:** Human Performance Research Group, Center of Health and Sport Sciences, Santa Catarina State University, 88080-350 Florianópolis, SC, Brazil

## Abstract

Lactate is a highly dynamic metabolite that can be used as a fuel by several cells of the human body, particularly during physical exercise. Traditionally, it has been believed that the first step of lactate oxidation occurs in cytosol; however, this idea was recently challenged. A new hypothesis has been presented based on the fact that lactate-to-pyruvate conversion cannot occur in cytosol, because the LDH enzyme characteristics and cytosolic environment do not allow the reaction in this way. Instead, the Intracellular Lactate Shuttle hypothesis states that lactate first enters in mitochondria and only then is metabolized. In several tissues of the human body this idea is well accepted but is quite resistant in skeletal muscle. In this paper, we will present not only the studies which are protagonists in this discussion, but the potential mechanism by which this oxidation occurs and also a link between lactate and mitochondrial proliferation. This new perspective brings some implications and comes to change our understanding of the interaction between the energy systems, because the product of one serves as a substrate for the other.

## 1. Introduction

Contrary to what previously thought, lactate is a glycolysis byproduct that can be produced and utilized continuously by various body cells, at rest, and even under conditions of adequately oxygenation [1–3]. It is a highly dynamic metabolite, and its shuttling through the interstitium and the bloodstream works as an important carbon source for oxidation in many tissues [4–7] or for liver gluconeogenesis [8, 9], especially in situations where an intense physical effort is required [10, 11]. In addition, it has been shown that lactate exposure decreases the activities of both the hexokinase and phosphofructokinase enzymes (PFK) and hence the muscle glycolysis in a dose-dependent manner [12, 13].

In the last few years, some studies have been published around lactate metabolism, mainly concerning its route of removal inside the cell, and a new hypothesis arose. Rather than the traditional view of Stainsby and Brooks [1], where lactate-to-pyruvate conversion occurs on the adjacencies of mitochondria, the intracellular lactate shuttle hypothesis (ILS) currently suggests that this chemical reaction takes place within mitochondria, more precisely in the intermembrane space [14, 15].

This new model requires the involvement of some proteins already known in the lactate dynamics, particularly the lactate dehydrogenase enzyme (LDH)—the protein responsible for the lactate oxidation and reduction—and the MCT1, one of the monocarboxylate transporter isoforms that are in charge for lactate transposing between tissues. The likely location of these two structures into mitochondria would allow that the lactate oxidation reaction and the subsequent transport of the newly formed pyruvate-to-the mitochondrial matrix could occur. Many studies support this hypothesis [16–21]. However, there is considerable disagreement about the presence of both proteins and hence lactate oxidation in the mitochondrial reticulum, mostly in skeletal muscle tissue.

Therefore, the aims of this review are to (1) expose the whole ongoing debate about the presence/absence of the ILS in key tissues that are exercise-related, (2) make a critical appraisal on lactate oxidation complex (LOX), probable mechanism by which lactate is used as an energy source by cells, and (3) comment on the recent discovery of lactate as a signaling molecule and also its presumed role in mitochondrial biogenesis. The knowledge about these issues is needed in understanding the energy supplies systems functioning and how they interact with each other to meet the body energy requirements.

## 2. Theoretical Background

Before delving more specifically on the theme proposed by this paper, some explanation is needed regarding the structure and operating of the major components which play a central role in the ILS: the LDH and MCT1.

## 3. Lactate Dehydrogenase Enzyme (LDH)

The LDH is a tetrameric enzyme, that is, comprised by four subunits. These smaller units could be, in turn, of two types: muscle (M) or heart (H). Therefore, the different possible combinations allow the existence of five isoforms (LDH1 to LDH5), all favoring lactate production [22]. Generally, exercise physiology or biochemistry books allude the direction of the LDH-mediated reaction (1) to the operating isoform [23–25], where M-prevailing isoforms would act primarily in lactate formation, while the isoforms that show H dominance would facilitate the reaction in the reverse direction. However, rather than this conservative view, it has been recently suggested that the cell compartmentalization and the LDH association with specific cellular structures (e.g., sarcoplasmic or mitochondrial reticulum) may play an important role in determining the lactate and pyruvate concentrations in each intracellular compartment, regardless the enzymatic isoform present [26].

(1) $$\text{Pyruvate}\;+\;\text{NADH}\;+\;{\text{H}}^{+}\;\to \;\text{Lactate}\;+\;{\text{NAD}}^{+}$$

This situation is feasible to occur, among other reasons, because LDH is a near-equilibrium enzyme; that is, it is not regulated allosterically or in a covalent manner, but instead by the concentrations of their substrates and products [22].

## 4. Monocarboxylate Transporter (MCT)

The lactate transport through different tissues is accomplished by a family of proteins termed monocarboxylate transporters (MCTs) [14, 27–31], which are stereospecific for L-lactate [32]. In such type of transportation, each molecule is carried throughout the membrane along with a hydrogen ion (H^+^), which gives to the MCTs a quite important role in pH regulation during exercise [28, 33].

Regarding lactate transposition, specifically, the several MCT isoforms seem to play slightly different functions in the human body. For instance, the MCT1 and MCT4 appear to be the most important conveyors in the exercise related lactate exchange among tissues, and it is particularly true in skeletal muscle. While MCT4 is apparently related with lactate efflux from highly glycolytic muscle fibers, the MCT1 has been associated to its uptake for further oxidation [14, 28, 30, 31, 34–36].

Just as the MCT family enables the lactate transfer between the many barriers of the body, these proteins are also capable to carry other forms of monocarboxylates across cell membranes, particularly pyruvate. This information shall be useful in the future when understanding the controversy generated due the presence of MCT1 on the mitochondrial reticulum.

## 5. The Intracellular Lactate Shuttle—Basic Concepts

The ILS was first proposed in 1998 in [10]. The general principle is based on the fact that the lactate to pyruvate conversion could hardly occur in the cytosol, once that (1) LDH has a high affinity for its substrate (i.e., a lower *K*_*m*_), (2) its V_max⁡_ is the greatest among the glycolytic pathway enzymes, and (3) its equilibrium constant favors the reaction toward lactate formation [17, 18]. In other words, how could the oxidation of an apprehended lactate molecule occur on cytosolic environment if all LDH characteristics favor the reaction on the opposite direction?

According to the ILS, lactate is an inevitable product of glycolysis which can be directly utilized as an energy source by mitochondria of various body cells, such as striated muscle cells and neurons, especially in situations of physical exercise. In addition, the ILS allows and helps to explain the functioning of the cell-cell lactate shuttle theory [11, 37], where the lactate mainly formed in cells with a high glycolytic capacity (e.g., type II muscle fibers) could be extruded to extracellular compartment and captured by both liver for gluconeogenesis or highly oxidative cells (e.g., type I muscle fibers) for further mitochondrial oxidation, mostly in subsarcolemmal mitochondria of muscle tissue [3].

Despite the mitochondrial reticulum being a common feature to almost all mammalian cells, we should be cautious about interpreting the different studies regarding its methods and tissues involved. Therefore, we chose to divide the topics by tissue and in some cases by structure, in order to facilitate the understanding and to better organize its current status in the literature.

## 6. Heart

Several studies have supported the view of mitochondrial lactate oxidation by myocardium. In 1988, Gertz et al. [5] were the first to demonstrate the increase in lactate consumption by cardiac muscle fibers during moderate exercise in humans. Thereafter, Laughlin et al. [38] infused isotopically labeled lactate tracers in the coronary circulation of dogs and detected its incorporation and utilization as energy source. However, and as stated by Brooks [39], no tracer appeared as either pyruvate or alanine forms, which would be expected if lactate oxidation was being accomplished in cytosol. In line with these studies, directly lactate oxidation was observed on both rat isolated mitochondria [18] and by magnetic resonance spectroscopy (MRS) in isolated perfused rat heart [40]. In addition, the metabolism compartmentalization was also showed in these cells, with glycolysis-derived lactate being preferentially expelled by the fiber, whereas the exogenous lactate absorbed and delivered to the mitochondria as potential energy.

Nevertheless, for this energy to be extracted and effectively used, it must have a mandatory lactate-to-pyruvate conversion when inside the mitochondrial reticulum. Indeed, some research has reported the presence of LDH in heart mitochondria [18, 41, 42]. These studies corroborated the initial findings of Baba and Sharma [16], the first to demonstrate the existence of the mitochondrial LDH (mLDH) on this tissue using combined techniques of immunohistochemistry and electron microscopy, which initiated the speculation on the involvement of this organelle in lactate's aerobic metabolism.

So far, only one study revealed to be contrary to the ILS in the heart, because it has failed to obtain expressive lactate oxidation in rat mitochondria *in situ* [43]. However, it was argued that the methodology of this work resulted in mLDH loss during sample preparation and that the low values of oxidation obtained reflected a small remnant amount in the intermyofibrillar mitochondria located in the deepest parts of the fiber [20].

Perhaps the real contradiction concerning the ILS in cardiomyocytes is the actual mechanism by which this mitochondrial oxidation occurs. In addition to the redemonstration of both the mLDH and mitochondrial lactate oxidation, Brooks et al. [17] have shown the presence of the mitochondrial MCT1 (mMCT1), a result which was later replicated [19]. This finding led to the assumption that this transporter would have a key role in lactate oxidation. On the other hand, it was also suggested that a system which involves a mitochondrial lactate/pyruvate antiporter (other than MCT) is responsible for controlling the mitochondrial importation of reducing equivalents [42]. However, these later findings have been associated with data misinterpretation [19].

Based on these findings, it seems quite reasonable to say that heart mitochondria are capable of oxidizing lactate directly, and although little discrepancy around the engine is involved, evidence points to MCT1 as an operative transporter in mitochondrial lactate oxidation in these cells. This later assertive will be somehow strengthened when analyzing the studies performed in other body tissues.

## 7. Neurons

Although the nervous tissue is not the main body site of removal, neuron cells could indeed use lactate as energy resource [7, 44]. Therefore, the conjecture about the ILS presence in the brain helps to support the already demonstrated astrocyte-neuron lactate shuttle [45], which could be summarized as an example of the cell-cell lactate shuttle but just between these two nervous cells.

Recently, it was shown that the rat cerebellar granule cells mitochondria are able to oxidize lactate directly, as well as the presence of the mLDH in the mitochondrial inner compartment by immunological analysis [46]. Moreover, it was observed, using eletrophoretic and immunohistochemical analysis, ^13^C-NMR, and HPLC, that mLDH acted in lactate oxidation in human astrocytoma cell cultures, demonstrating for the first time the ability of astrocytic mitochondria of consuming this metabolite [47]. These works served as the basis for the most recent findings of Hashimoto et al. [21], which provided *in vivo* and *in vitro* evidence of the LOX—the current model of lactate oxidation by mitochondria (but it can wait until later)—and thus the ILS in rat brain, more specifically in hippocampus, thalamus, and cortex. Nevertheless, beyond the MCT1, the authors also reported the presence of the MCT2 isoform in the mitochondrial reticulum. Given this somehow unexpected novelty, they conclude that the LOX might present itself under two forms: with MCT1 and MCT4. This variation could be very important, since that both isoforms have distinct affinities by the substrate and hence allow some flexibility to attend the changes in the extracellular concentrations of lactate which takes place in different situations.

## 8. Skeletal Muscle

Skeletal muscle is the major site of lactate production and removal of the body. In several types of exercise, lactate is released in the bloodstream only transiently and, after a given time period, a shift occurs and active muscle starts to consume, rather than produce, lactate as an energy resource [6]. This situation occurs mainly in type I and IIa muscle fibers, where the main fate of lactate is oxidation (on the type IIx muscle fibers its primary route of removal is by glycogenesis) [6, 48]. This is particularly true during sustained submaximal exercise, when the blood lactate concentrations are above rest values, making an ideal concentration gradient for lactate uptake [49]. In addition, some other factors determine the intensity of lactate consumption by working muscles, such as muscle metabolic rate, optimal levels of intra- and extracellular pH, adequate blood flow, and fitness level [50].

In this particular tissue, the ILS is very polemical. Because it is an especially contentious topic, we chose to split it into its main parts. Firstly, the studies that have attempted to obtain significant mitochondrial respiration from lactate and sought to locate the mLDH in the mitochondrial reticulum will be present. Thereafter, we will depict the discussion about the MCT isoform that acts in this process, and, ultimately, we will expound the LOX, presumed mechanism by which lactate oxidation occurs into mitochondria.

### 8.1. Mitochondrial Lactate Oxidation and mLDH

There has been a huge debate in the literature as to whether mitochondria contain an LDH isoform and perform direct lactate oxidation. The investigation of a putative LDH within mitochondria relies basically on two approaches: (i) isolation of a mitochondrial fraction free of other cellular constituents followed by respiratory and enzyme activity assay and (ii) tissue histology followed by colocalization of mitochondria and immunolabeled LDH. Each of these methods has its intrinsic pitfalls that may account for the discrepancy between the studies reviewed below.

Lactate oxidation by rat skeletal muscle has been evidenced through the analysis of carbon-labeled substrate metabolism. These data suggested that lactate conversion to pyruvate takes place within mitochondria rather than in cytosol [51]. On the same year, Brooks et al. [18] showed the oxidation of exogenous lactate in isolated rat mitochondria, as well as the occurrence of an mLDH on this tissue using gold particle immunolabelling and electron microscopy. In addition, the mLDH isoforms and their distribution pattern were different from those LDH found in neighboring cellular compartments, which was argued to rule out any likely contamination with cytosolic isoforms (cLDH). Similarly to heart mitochondria, this work has confirmed the earlier findings by Baba and Sharma [16], regarding the presence of mLDH in skeletal muscle, and those of Nakae et al. [52], who demonstrated the colocalization of LDH with the mitochondrial enzyme succinate dehydrogenase in mice, and hence have led to the conclusion by Brooks et al. [18] that lactate is the major monocarboxylate oxidized by skeletal muscle mitochondria *in vivo*, mainly when the lactate/pyruvate ratio is high, as in physical exercise.

Thereafter, a number of papers challenged this concept of ILS, and several concerns about mitochondrial constituents loss and sample contamination by cytosolic components were raised. Firstly, Rasmussen et al. [53] and Sahlin et al. [54] did not observe mitochondrial respiration with lactate as substrate in organelles isolated from rats and humans skeletal muscle; thus suggesting that the results of Brooks et al. [18] were possibly an artifact of a contamination with cLDH, which could have remained in the mitochondrial reticulum during the sample preparation. In response, Brooks [39] provided theoretical arguments favoring the ILS and claimed that these contradicting findings could be due to methodological differences such as the use of proteases in the mitochondrial isolation process, which would have resulted in mLDH loss during preparation. This latter statement was readily contested by some studies [55, 56], which made reference to the work of Ponsot et al. [43] with skinned muscle fibers, where no protease was used and still no significant mitochondrial lactate oxidation occurred. However, this assertion was rebutted by Brook's group, and the latter result associated this time to the saponin treatment, which also led to mLDH loss during the preparation procedures [20].

Additionally, there were other arguments quoted by Yoshida et al. [56] in an attempt to refute the ILS concept. They used the study of Szczesna-Kaczmarek [57], the first to show the direct mitochondrial lactate oxidation (and another one that showed mLDH in rat muscle), but in a following work with humans they attributed their earlier findings to contamination [58]. In addition, Yoshida et al. [56] have provided the main barrier against the ILS. They isolated highly oxidative and glycolytic muscle fibers (gastrocnemius and tibialis anterior) from rats and compared its lactate and pyruvate oxidation rates. They also investigated whether there is any difference in metabolic capacity between the two mitochondrial subpopulations (subsarcolemmal and intermyofibrillar) in an attempt to justify the findings of Brooks et al. [18]. Among the results of Yoshida et al. [56] are (1) negligible lactate oxidation by both mitochondrial populations (1/31th to 1/186th of that pyruvate), (2) the extremely low activity of LDH (1/200th to 1/240th of that from whole muscle homogenates), and (3) addition of exogenous LDH promoted lactate oxidation. According to the authors, the use of pyruvate at much higher levels than lactate would be consistent with previous studies [43, 53, 54, 58] and reflects the absence of mLDH and hence the ILS.

Indeed, it is interesting that no other group has been able to successfully replicate the results of Brooks et al. [18]. Therefore, it was insistently suggested that the results of this study could reflect either some degree of contamination by cytosolic LDH or the oxidation of malate, rather than lactate [55, 56]. Nevertheless, these hints were rejected by Brooks and Hashimoto [59]. They stated that the presence of the necessary structures for the ILS was showed several times and with a variety of methodological approaches. In addition, they suggested that the protease used by Yoshida et al. [56] (Subtilisin A) culminated with the loss of the ILS fundamental components: mLDH and MCT1.

Probably, the protease treatment is really the culprit for the disagreement about the ILS on skeletal muscle. Recently, another research group claimed to reproduce the results of Brooks et al. [18] in a preliminary assay [60]. Using rabbit gastrocnemius mitochondria, there was an increase in the fluorescence of the intramitochondrial NAD(P)H. In parallel, they also reported the occurrence of the mLDH. Remarkably, none of these outcomes could be reproduced when the protease was used. Regarding these protease issues, it may be argued that the putative loss of mLDH upon tissue processing in the presence of protease means that mLDH is located in a cellular compartment readily accessible to the protease, that is, outside inner mitochondrial membrane.

The MitoCarta available at http://mitominer.mrc-mbu.cam.ac.uk/ indicates proteomic studies that have identified LDH as a human mitochondrial protein. However, this is not the end of the dispute because the proteomic approach is only as good as the intactness and purity of the analyzed samples (i.e., isolated mitochondria). Therefore, some concerns around the respiratory and enzymatic assays stem the proteomic approach as well. Nevertheless, it is likely that skeletal muscle mitochondria are also capable of directly oxidize lactate. However, in our point of view, and of others [61], only the methodological advancements related to the mitochondrial isolation will put an end to this discussion.

### 8.2. Mitochondrial Monocarboxylate Transporter and the Lactate Oxidation Complex

Brooks et al. [17] located the mitochondrial MCT (mMCT) in the two mitochondrial subpopulations of rats and found that it was the MCT1. Afterwards, a careful study replicated this finding [19]. These results provided support for those of Dubouchaud et al. [62], which assessed the effects of nine weeks of training in the vastus lateralis muscle of sedentary subjects. They found an increase in the mitochondrial MCT1 content, as well as a higher activity of mLDH (associated with an elevated mitochondrial mass), strengthening the idea of the ILS also in humans.

On the other hand, other studies found very different outcomes [56, 63]. Therein, the MCT1 and MCT4 isoforms were located in subsarcolemmal, but completely absent in intermyofibrillar rat mitochondria, while MCT2 was present in both subpopulations. Based on these results, Benton et al. [63] suggested that mitochondrial subpopulations might have different metabolic capabilities and that if mitochondria really could oxidize lactate, it would be probably the subsarcolemmal class.

With the aim of analyzing the discrepancies of the aforementioned studies, Hashimoto et al. [14] used confocal laser scanning microscopy (CLSM) to examine the intracellular domains occupied by the MCT in different muscle fiber types of rat skeletal muscle. As result, only weak signals for MCT2 were found (in the sarcolemma), whereas MCT1 was present throughout the interior of type I and IIa muscle fibers, and in both subsarcolemmal and intermyofibrillar mitochondria. Subsequently, these results were further strengthened [20]. In addition, they commented the both works opposing the notion of MCT1 as operating mitochondrial monocarboxylate transporter. To them [14, 59], Yoshida et al. [56] and Benton et al. [63] ignored strong evidence that mitochondria exists in a reticulum form—and it is actually true [64]—and that the methodology employed by them resulted in mitochondrial disruption and hence in the loss of some mitochondrial constituents and functions, as well as in sample contamination by MCT isoforms pertaining to other cellular structures.

At this point, the notion of MCT1 as the mMCT became stronger and the studies have taken a new course. For instance, Hashimoto et al. [20] used combinations of stringent confocal scanning microscopy, immunoprecipitation, and western blotting after cell fractionation to demonstrate the colocalization of the LDH, the MCT1, and the chaperone protein CD147 (basigin)—a protein that supposedly anchors MCT1—with the mitochondrial reticulum of cultured L6 cells. The Western Blot method showed plenty of NADH dehydrogenase (NADHDH), cytochrome oxidase (COX), LDH, MCT1, and CD147 in the mitochondrial fractions of these cells, and the immunoprecipitation method showed interactions with COX, MCT1, and CD147. These novel results have extended those previously showed [14], where it was already demonstrated the colocalization of MCT1 and COX in both mitochondrial domains, and thus enabled the group to propose the existence of a mitochondrial lactate oxidation complex (LOX), associated with the complex IV of the electron transport chain on the inner mitochondrial membrane, which involve the proteins MCT1, mLDH, CD147, and COX (for a full schematic view, see [20]).

According to the LOX model, the high cytosolic concentration of lactate, lactate/pyruvate, and H^+^—as well as the high lactate/pyruvate ratio in this compartment—are juxtaposed with its lower concentrations in the mitochondrial matrix. Additionally, in contrast to the changes observed in the cytosol, the electron transport chain is relatively more oxidized during exercise. These factors facilitate the lactate influx and hence mitochondrial oxidation, because the environment favors the pyruvate and H^+^ removal via the citric acid cycle and mitochondrial ATPase, respectively. Besides, and as mentioned earlier, LDH characteristics do not favor the cytosolic lactate oxidation. So, the model mechanism proposes that lactate is oxidized by mLDH, which is possibly anchored to the outside of the inner mitochondrial membrane in association with COX. This endergonic reaction seems to be coupled with the exergonic redox change on COX during the mitochondrial electron transport. In other words, the oxidizing environment of COX favors the lactate-to-pyruvate conversion. Once the reaction has occurred, the newly formed pyruvate molecule could leave the intermembrane space where it was formed and be transported through MCT1 to the mitochondrial matrix, to be catabolized by the cellular respiration process [15, 20, 37].

In summary, based on the aforementioned studies conducted in skeletal muscle tissue, we can conclude that (1) lactate can indeed be oxidized by mLDH in skeletal muscle mitochondria and (2) the oxidation process employs the MCT1 on the subsequently transport of pyruvate into mitochondrial matrix. Despite being a relatively new model, supported by only a few studies, the LOX consists in the probable mechanism by which skeletal muscle consumes lactate and thus helps to explain why training increases lactate removal by active muscles and also exert a role in lactate accumulation during exercise [20, 65]. The presence of the LOX on mitochondria is quite reasonable since another strong point is that its structures have being reported in studies of the mitochondrial proteome [66, 67].

## 9. Lactate Role in Mitochondrial Biogenesis

After the LOX proposal, Hashimoto et al. [68] investigated the effects of lactate exposure in L6 cells cultures and found that the contact with the metabolite, as well as the increase in the O_2_ consumption, leads to the generation of reactive oxygen species (ROS), such as hydrogen peroxide (H_2_O_2_). As a consequence, an event cascade begins, raising the expression of several genes related to mitochondrial biogenesis and thereby elevating the mitochondrial mass. Furthermore, lactate exposure increased all LOX-related proteins. In addition, it has been also shown that mitochondrion exist as an elaborated and dynamic network, where each mitochondria exists individually only transiently. Therefore, the balance between the fusion and fission rates defines the actual morphology of the whole reticulum [64]. On this merit, Fan et al. [64] recently demonstrated that H_2_O_2_ exposure influenced temporarily these indexes and thus suggested that oxidative stress may contribute to the exercise-induced in mitochondrial morphology *in vivo*.

Taken together, these findings of lactate-mediated genetic stimulation and acute mitochondrial morphology modification demonstrate an important role in cell signaling performed by lactate, and, therefore, a term of lactormone has been used as an epithet, since it can affect the expression of transport proteins, mitochondrial biogenesis, and other adaptive responses generated by exercise stress [15, 69].

## 10. Conclusion

In summary, lactate seems to be the main monocarboxylate oxidized by the mitochondrial reticulum *in vivo*, especially in situations where the lactate/pyruvate ratio is high, such as during an intense physical effort. Furthermore, it acts as a kind of “bargaining chip” between tissues, a moving glycogen, which is able to provide raw material for ATP resynthesis in mitochondria of a wide variety of body cells. The ILS enables and explains how this mechanism occurs. Through the ILS, this valuable metabolite can be directly imported by the mitochondria of the most important tissues involved in the exercise and then be oxidized by the LOX, at least in the striated and nervous tissues, where the existence of such machinery has been demonstrated. This situation implies that much of the formed lactate will be oxidized without ever leaving the muscle, and even more, without ever leaving the producing cell. Furthermore, it has been shown that lactate exposure leads to ROS generation and that this oxidative stress is responsible for (1) activating several genes favoring mitochondrial biogenesis and (2) may even influence the morphology of the entire mitochondrial reticulum, as it interferes in both mitochondrial fusion and fission rates.

Particularly in skeletal muscle, the ILS comes to change our understanding of the energy systems as well as the interaction which occurs between them. In this new view, rather than two relatively isolated energy systems, they can be seen as two independent parts of the same interconnected system, because the endpoint of one serves as a fuel for another [37, 70]. As the rates of ATP supply from these systems are distinct and differently regulated, the much faster initial part of the route (i.e., anaerobic glycolysis) ends up being of specific benefit in both the beginning of exercise or when exercising in intensities at which the oxidative phosphorylation cannot account for the whole rate of energy release.
